# Skeletal Muscle Memory: An Update From the Antidoping Perspective

**DOI:** 10.1002/dta.3804

**Published:** 2024-09-24

**Authors:** Claire Traversa

**Affiliations:** ^1^ World Anti‐Doping Agency (WADA) Montreal Quebec Canada; ^2^ Department of Kinesiology and Physical Education McGill University Montreal Quebec Canada

**Keywords:** antidoping, anabolic androgenic steroids, doping, muscle anabolism, muscle hypertrophy, muscle memory, myonculei density, myonuclear domain theory, myonuclei

## Abstract

This narrative review explores the concept of muscle memory, focusing on the physiological and biochemical mechanisms underlying information retention in skeletal muscle tissue as it relates to antidoping. The discussion encompasses the role of satellite cells (SCs) in myonuclei recruitment, resulting in increased myonuclear density and heightened muscle protein turnover. The myonuclear domain theory suggests that myonuclei acquired during hypertrophy may persist, contributing to enhanced muscle protein synthesis (MPS) and potential benefits of muscle memory. The impact of sustained training, protein intake, and resistance exercise on muscle memory, especially in elite athletes, is considered. The review also delves into the influence of anabolic androgenic steroids (AAS) on muscle tissue, highlighting their role in elevating the performance threshold and supporting recovery during intense training through increased muscle protein turnover rates. Additionally, genetic and epigenetic modifications, such as DNA methylation, are explored as potential contributors to muscle memory. The complex interplay of continuous training, AAS use, and genetic factors offers avenues for further research, especially in the context of antidoping efforts. The understanding of muscle memory has implications for maintaining performance gains and addressing ethical challenges in sports.

## 1 Introduction: Defining Muscle Memory

Memory is the process by which information is stored, encoded, and retrieved when needed [1]. By nature, we often think of these processes as being done exclusively by the brain, though more recently, the cells have shown similar capabilities in other tissues. Muscle cells are the largest cells in the human body, housing multiple nuclei—referred to as myonuclei at the muscle level [1]. These nuclei are evenly distributed throughout a muscle fiber [2]. During physical activities or the repetition of motor tasks, the body relies heavily on the central nervous system (CNS) to remember the details of performing these tasks in order to be able to repeat them unconsciously (i.e., climbing stairs or kicking a soccer ball). This creates what is often referred to as a mind–muscle connection recruiting both the unconscious and procedural memory of the CNS to perform daily tasks. In the CNS, this manifests physically as an increased number of synaptic connections or white matter in the brain, increased spinal motor neuron excitability, or induction of synaptogenesis within the spinal cord [3, 4]. At the muscle level, repetition is believed to create a physical imprint by increasing myonuclei, referred to as myonuclear density. In the general population, this neurological and physiological muscle memory applies to everyday tasks such as riding a bicycle or carrying a large bag of groceries. Athletes, on the other hand, experience this phenomenon in sport‐specific skills (e.g., spiking a volleyball or executing a golf swing) and when reaching peak physical performance [2]. Recently, discussions have emerged regarding how muscles retain information under the influence of anabolic androgenic steroids (AAS). These conversations became more prominent when we began seeing athletes who had served a doping violation return to the Olympic Games or international competition, outperforming themselves prior to their sanction. The intention of this perspective update is to define muscle memory as it applies to the athlete population and how it may be affected by the use of AAS.

## 2 Satellite Cells

SCs were given their name after being discovered to reside on the sarcolemma boundary of the muscle fiber [5, 6]. They first became known for their role in muscle regeneration [5, 6, 7]. Further research confirmed their involvement in many cellular processes including those related to muscle adaptation [5, 8, 9]. Recently, researchers have taken a keen interest in understanding how SCs contribute to muscle hypertrophy and the accumulation of myonuclei [10]. SCs habitually lay dormant on the basal lamina, only to be interrupted by activation in response to the muscle's strenuous demands [5, 11]. Once activated, these cells undergo proliferation and differentiation through cell division, a process known as myogenesis [5, 12]. Following a stimulus such as muscle damage or inflammation resulting from heavy resistance exercise, SCs will translocate to the site of damage, bind to existing muscle fibers, and differentiate to contribute as new myonuclei [5, 11]. In fact, SCs are the main source of new myonuclei in the muscle fiber [5, 12]. Worth noting, recruiting myonuclei becomes more challenging in elderly individuals compared to young, healthy populations, emphasizing the importance of initiating strength training from a young age to establish a robust myonuclei library [5]. Once recruited, myonuclei can remain stable for up to 15 years [5].

## 3 The Myonuclear Domain Theory

**FIGURE 1:**
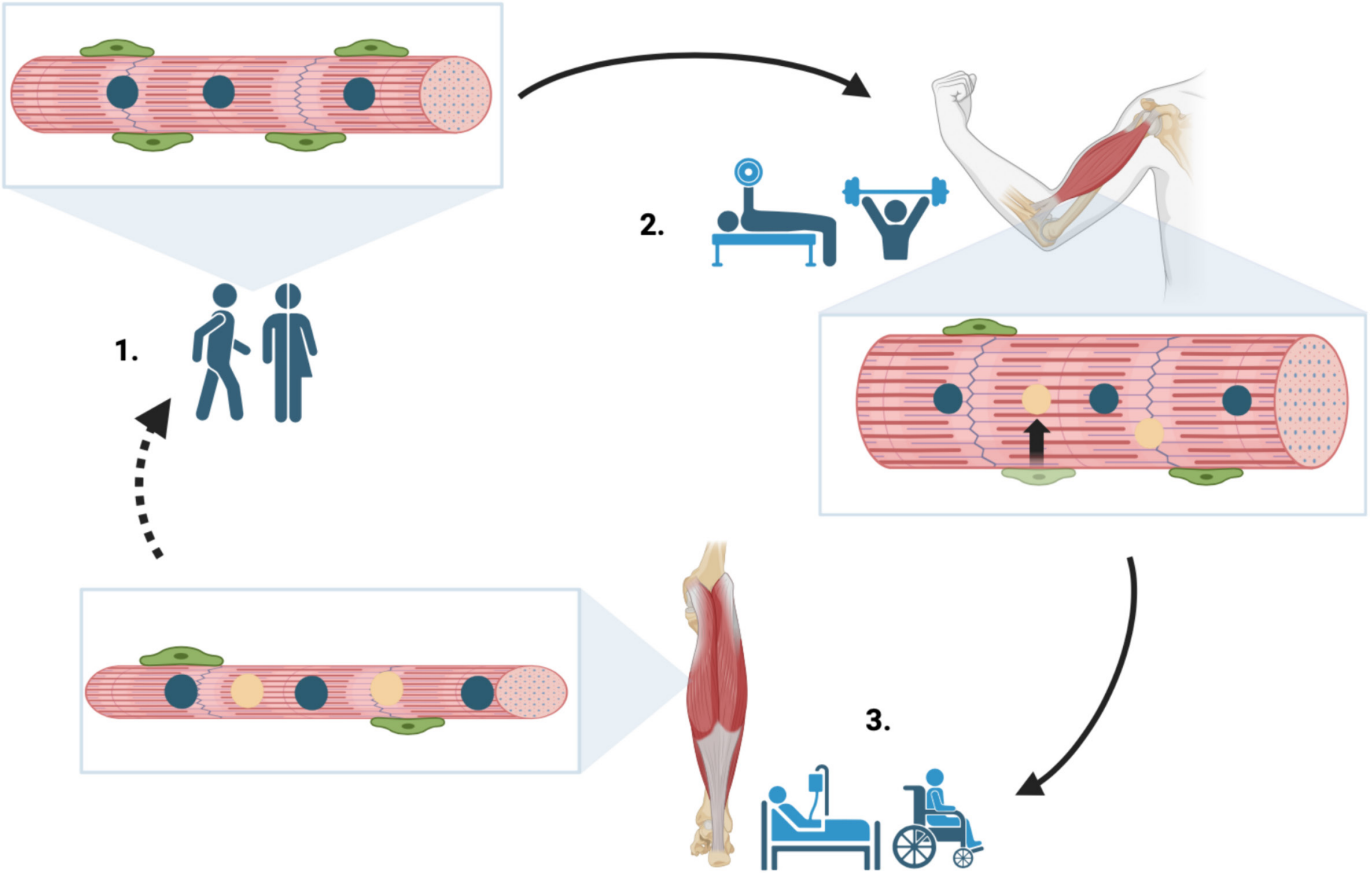
Schematic of the myonuclear density theory. 1. Skeletal muscle fiber (red cylinder) containing SCs (green) on the perimeter of the basal lamina and existing myonuclei (blue) in healthy active humans. 2. Satellite cells will be recruited as additional (new) myonuclei (yellow) during muscle hypertrophy phases such as through resistance exercise training. This produces a larger (hypertrophied) muscle fiber with increased an overall myonuclei number. 3. Following an episode of muscle atrophy (via injury or illness etc.), the result is a smaller muscle fiber with the retained number of myonuclei – covering a smaller area of muscle tissue. Created in BioRender.com.

The theory of myonuclear domain, initially introduced by Qaisar and Larsson in 2014, explains that each myonucleus in muscle fibers is believed to serve a limited “domain” of the cytoplasm for mRNA transcription that it does not extend beyond [13]. Through resistance training and other forms of exercise that promote skeletal muscle hypertrophy, activated SCs will be recruited from the muscle fiber perimeter and undergo myogenesis to become additional myonuclei [13, 14, 15]. On a grand scale, this will increase the transcriptional ceiling of the muscle fiber, elevating the rate of muscle protein synthesis (MPS) and ultimately resulting in an augmentation of muscle fiber size (hypertrophy) [1, 16]. Ongoing resistance training and skeletal muscle hypertrophy poses continual transcriptional demands on the muscle fiber, forcing the recruitment of additional SCs to increased myonuclei content, thereby decreasing domain size (due to a higher myonuclei:cytoplasm ratio) [1]. The physical indication of muscle memory at this level became evident as several studies saw that even after a period of muscle atrophy, myonuclei appeared to be protected from apoptosis, maintaining a higher myonuclei density per muscle fiber compared to those untrained (Figure 1) [15, 16, 17]. This suggests that increased myonuclei might play a role in maintaining muscle mass and homeostasis during periods of inactivity, though additional research is required for confirmation [15, 16, 17].

If increased myonuclei represents muscle tissue's way of storing and encoding memory, the next question is: “can muscle memory be retrieved when needed?” Animal models demonstrate that when rodents (mice) are re‐exposed to resistance training, fibers with higher myonuclei content resulting from preceding periods of resistance training exhibit faster growth during overload exercise compared to untrained animals undergoing resistance training for the first time [14, 16]. Again, this is attributed to the preservation of myonuclei, even during prolonged periods of detraining, and the idea that new myonuclei are recruited only when the muscle fiber experiences growth beyond its previous limits [15, 16]. Indeed, in the retraining process, it is thought that new myonuclei are only recruited when the muscle fiber is subjected to growth beyond the previous ceiling—otherwise, myonuclei density will remain consistent and new myonuclei will not be recruited [1, 14]. When this ceiling is exceeded, the increased number of nuclei expand the capacity for protein turnover [1]. The process of total protein synthesis is a product of synthesis per nucleus and number of nuclei [1]. We can look at this as a positive feedback loop as the greater the hypertrophy stimulus is, the greater the push on the transcriptional threshold is, and once increased, it allows for additional muscle growth to provide an even greater hypertrophy stimulus. Factoring in the use of anabolic agents on top of this further exacerbates the hypertrophy stimulus, which will be discussed later in this review [18].

## 4 The Effect of Resistance Exercise Alone

A single‐bout of damaging acute eccentric exercise (i.e., 10 sets, 30 repetitions at 180°/s) can elicit an increase in SCs and an activation of SCs, becoming apparent at 24 h and reaching a peak at 72 h during postexercise recovery [5, 18, 19]. Other studies in humans have shown that 3 months of resistance training (3 times/week) was sufficient to increase skeletal muscle fiber size and SC [20]. The 27% increase in SCs persisted for 10 days after complete exercise termination and only after 90 days of inactivity did SC number return to baseline levels [20]. Many studies have consistently demonstrated that skeletal muscle fiber hypertrophy is linked to increased SCs and myonuclear content during prolonged resistance exercise training, which is typical in elite athletes' training regimens [5, 17, 20, 21, 22, 23, 24]. During hypertrophy‐focused resistance exercise, SCs have been seen to increase concomitantly with muscle fiber area increases, regardless of fiber type, implying that myonuclear content is more closely associated with fiber area increases than fiber dominance during exercise [23, 24]. Mackey et al. (2007) first evaluated the myonuclei density theory in humans using a unilateral lower limb model involving a 12‐week resistance training period, followed by 20 weeks of detraining, and then a 5‐week bilateral retraining period. Surprisingly, myonuclei number and fiber volume were unchanged after the original training period, despite an increase in overall muscle size [25]. The authors suggested that this may be due to the initial training period not being an adequate stimulus to activate SCs and therefore establish cellular memory [25]. Similarly, Psilander et al. (2019) evaluated myonuclei accretion in humans using a unilateral model with 10 weeks of single‐leg resistance training, followed by 20 weeks of detraining, and then a 5‐week retraining program [26]. Despite increases to the trained leg in both muscle thickness and cross‐sectional area (CSA) following the original training program, there was no evidence of increased myonuclei in the trained leg [26]. Again, an insufficient increase in muscle fiber size was thought to be the reason for this observation [25, 26]. Myonuclei addition was then deemed to occur only in hypertrophy levels >20% (increase in CSA), whereas this study saw an increase of <10% [26]. This aligns with the theory of myonuclear domain, emphasizing that the transcriptional threshold must be exceeded quickly, aggressively, and for a sustained period of time to induce SC proliferation, differentiation, and eventual myonuclear accretion [5, 24, 25, 26]. Hence, SC activation is largely dependent on exercise type, volume, and/or intensity which warrants more research for confirmation of how long it may be necessary to induce these changes [24].

Previous research indicates that aging has a profound effect on the regulation of SC number and function in human skeletal muscle potentially challenging the muscle memory theory [27]. Sharples et al. (2023) proposed that if repeated hypertrophic stimulus exposure generates a physiological imprint, a similar phenomenon may exist for muscle atrophy (i.e., through recurrent injury or repeated periods of muscle wasting due to factors such as bedrest, illness, or disuse that result in the muscle being more susceptible to the same net loss of fiber CSA) [16]. It is well‐established that the process of muscle atrophy occurs much faster than muscle hypertrophy to change muscle fiber volume [15, 28]. This natural experience highlights a potential disadvantage of in vitro and animal models of muscle memory, as they do not consider repeated periods of injury or muscle wasting that humans inevitably face during their lifetime [29]. These atrophy periods considered the primary stimulus of sarcopenia onset and age‐related muscle wasting to which a period of detraining often seen in animal muscle memory studies is not accurately representative of, nor does it mimic the lifestyle of an elite athlete, which is the main focus of this review [29].

## 5 Anabolic Androgenic Steroids

At least 120 athletes with doping offences or sanctions for doping violations competed at the 2016 Olympic Games in Rio [26, 30]. Of these athletes, 31 individuals won medals [26, 30]. Notably, eight of these 31 medal winners were powerlifters who had been suspended for Anti‐Doping Rule Violations related to AAS from March 2013 to October of 2015 [31]. AAS exhibit both androgenic and anabolic effects, by mimicking masculine hormones (androgens) while promoting growth (anabolic effects) [32]. This occurs when the steroid binds to androgen receptors located in the cytoplasm of muscle cells. The steroid‐androgen complex will move inside the cell nucleus and bind to DNA, stimulating gene promotion for proteogenesis in myonuclei via increased rates of transcription and translation [32]. This process increases the density of muscle contraction proteins (actin and myosin), enhancing muscle power and strength output [32]. The insulin‐like growth factor 1 (IGF‐1) gene is also expressed during this time as it plays a key role in muscle growth and androgen receptor signaling [32]. This upregulation in androgen receptor signaling is what drives the physiological effects of steroids—by increasing the production of androgens and therefore the density of muscle proteins that can be transcribed and contribute to muscle growth [32, 33]. Both testosterone and AAS can bind to androgen receptors to promote additional receptor expression [32, 33]. The efficacy of steroid use is enhanced by unbound androgen receptor sites in skeletal muscle—a condition intensified by intense resistance training, doubling down on hypertrophic stimuli [32, 33]. SCs are also known to be a direct target of AAS circulating in the bloodstream, as they possess the androgen receptor [18]. This is why an increase in SC number is typically seen with current and past AAS users [22, 24, 34, 35]. When a supraphysiological dose of AAS is combined with resistance training over a consistent period of time, it creates an ideal hypertrophy stimulus to induce myonuclei accretion [16].

Muscle hypertrophy is the product of MPS being greater than muscle protein breakdown (MPS > MPB). As mentioned above, total protein synthesis is a product of synthesis per nuclei and number of nuclei (which can therefore be increased by SCs differentiating as additional myonuclei) [1]. The increase in myofibrillar protein synthesis is mediated by mTORC1 (the mechanistic target of rapamycin) complex 1, coupled with nutritional availability [36]. mTOR associates with accessory proteins which further phosphorylate S6 kinase 1 (S6K1) and eIF4E binding protein 1 (4E‐BP) to control the rate of protein synthesis [36]. Amino acid availability (particularly leucine) following a meal will induce secretion of insulin and IGFs to activate mTORC1 through the PI3K‐Akt signaling pathway [36]. Adequate abundance of both amino acids and transcriptional architecture such as myonuclei are crucial for muscle anabolism [36]. Considering athletes likely consume a higher‐than‐average protein diet (and therefore an abundance of amino acids), the combination of continuous resistance training, AAS use, and high protein intake may amplify SC activation and myonuclei accretion, leading to consistently elevated MPS rates. Resistance exercise and AAS use have been present in the recruitment criteria for previous muscle memory studies as their combination greater increases the chance at inducing myonuclei accretion in humans than when applied alone [18, 22, 34]. However, reflecting back on the typical behaviors of an athlete, does adding an abundance of nutrient availability to the cellar pathways that drive MPS further increase the likelihood of developing muscle memory framework. This warrants further investigation to be consistently relevant to elite athletes.

Muscle memory or myonuclear permanence explains that the retention of additional myonuclei acquired during a hypertrophic stimulus may persist following detraining, muscle wasting, and/or cessation of AAS use [26, 37]. Multiple animal studies support this, suggesting that a snapshot of myonuclei density at any given time represents the largest the muscle fiber has ever been rather than its current size or transcriptional capacity [37]. As of now, this is the only known physiological imprint that could be detected in a previous user of AAS (i.e. once [16, 18, 37, 38]. This becomes advantageous for athletes as the hypertrophic ceiling in previous AAS users is likely higher than that of a natural athlete who has never used AAS [18, 37, 38]. The increased myonuclei density in a previous AAS user would allow them to return to their peak muscle mass sooner, following a period of detraining or muscle loss [26]. What remains unclear is whether an athlete who has a history of AAS use but no history of detraining or athletic downtime still reaps the threshold‐extending benefits of myonuclear permanence following cessation of use. In other words, whether athletes sanctioned for 2–4 years, confirmed AAS‐free but engaging in other anabolic stimuli such as resistance exercise and protein intake, experience the transcriptional benefits of myonuclear permanence. The only group to investigate this so far was Nielsen et al. (2023) who noted that former AAS users that maintained their training regime still experienced a decline in muscle CSA despite displaying a higher myonuclei density [18]. This implies that blunted MPB rates experienced during AAS use returned to “normal” levels upon cessation resulting in the net loss of muscle proteins (and muscle mass) [18]. The exact training history of the participants in this study is unknown as they are described as recreationally resistance exercise trained making it unclear for how this experience may relate to that of elite athletes [16].

The Gundersen laboratory investigated the myofibrillar effects of testosterone administration on muscle memory in mice. They subjected mice to 14 days of testosterone treatment to promote muscle hypertrophy and myonuclei accumulation followed by a 3‐month washout period without testosterone [38]. Subsequent mechanical overload synergist muscle ablation reintroduced a hypertrophy stimulus resulting in a 31% increase in muscle fiber CSA in the testosterone group compared to a 6% increase in the control group [38]. This is one of the first animal models to display the “retrieval” effect of myonuclear permanence following the administration of AAS and a period of muscle atrophy, which, although valuable, still requires confirmation in humans [38].

**FIGURE 2:**
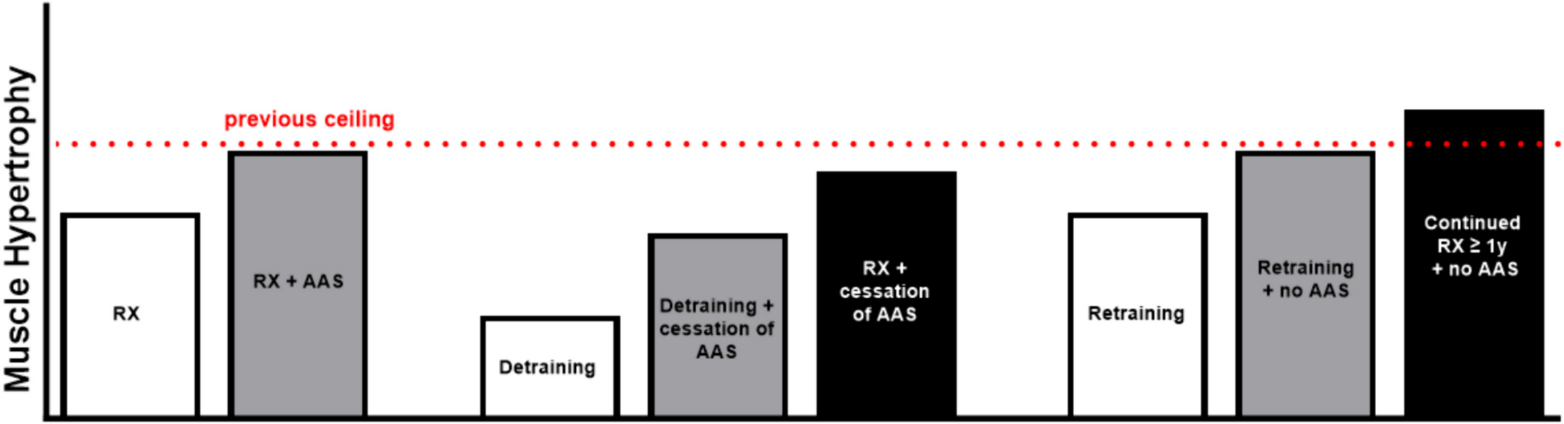
Hypothetic scenarios linking resistance exercise training and AAS use: the white bars represent a natural/clean athlete; where the left bar RX = Resistance exercise training period, followed by a decrease in muscle hypertrophy coinciding with a period of detraining in the middle, and finally an increase in muscle hypertrophy during a period of retraining at the far right. The gray bars represent the training cycle of an athlete that is caught for AAS use and undergoes a period of detraining before retraining. It is hypothesized that their muscle hypertrophy level will drop at the cessation of AAS use and detraining but will increase back to the previous level upon retraining. Finally, the black bars represent an athlete who may have been sanctioned for doping and stops doping, but never stops training. The muscle hypertrophy levels for this athlete are expected to drop immediately after cessation of AAS but increase back to and potentially exceed the previous ceiling with continued resistance exercise training. The previous ceiling created while being resistance trained and using AAS is highlighted in red.

Figure 2 visually displays the latter hypothesis, where there is an assumption that high performance athletes will consistently train and condition their muscles for performance improvement, while some of these athletes may potentially use AAS on top of this. Myonuclei can remain stable for up to 15 years and may even become permanent, typically representing the largest the muscle fiber has ever been [13]. The myonuclei pool has been seen to be resilient through many different muscle wasting conditions such as general atrophy, cancer cachexia, denervation, and sarcopenia [18, 39]. This suggests that following cessation of AAS, a decrease in skeletal muscle fiber size may occur, but SC activity, myonuclear density, and neurological connectivity remain. Second to this, for many athletes, two other hypertrophy promoting inputs will remain despite cessation of AAS:resistance training and protein intake. This raises the question fate of a former AAS user who never experiences a period of detraining. Most researchers investigating the muscle memory phenomenon have included a period of detraining or unloading, which may not align with the continuous training athletes will undergo. Therefore, the impact of sustained training on muscle memory warrants further investigation to better understand how muscle memory can be harnessed in the context of antidoping efforts.

## 6 Anticatabolic Effects of AAS

Apart from inducing anabolic effects, AAS have also been shown to prevent MPB and protect the muscle from wasting [32, 33]. Exercise‐induced cortisol production stimulates proteolysis (protein breakdown) and initiates gluconeogenesis, a process where amino acids bound in proteins break down to provide energy for metabolism [32, 33]. This depletes the free amino acid pool available for proteogenesis (protein building) precursors [32, 33]. Glucocorticoid receptors are found in skeletal muscle and allow both testosterone and its steroid mimic to bind, blocking the binding of cortisol to prevent muscle atrophy [32, 33]. This process not only protects acquired muscle tissue but also shortens recovery time by allowing amino acid precursors to be readily available for tissue repair and regeneration. This may further protect myonuclei acquired during hypertrophy from being lost during any muscle wasting after substance cessation. Another way this has been seen as advantageous to previous AAS users is in a more recent publication by the Danish group who observed that former users maintained a higher percentage of lean body mass compared to natural resistance trained controls even years after cessation of AAS use [18].

In terms of performance and strength, many researchers have recorded measurements such as 1 repetition maximum tests (1RM), peak power output, and similar indicators of strength. The most recent publication from the group in Denmark saw that previous AAS users had significantly higher 1RM values for standard strength tests including bench press and leg press [18]. In addition to maintenance of greater strength test scores after cessation of AAS use, greater myonuclei density also remained in these athletes [18]. Unsurprisingly, increased duration of AAS use was associated with increased myonuclei density while time since cessation of use was not [18]. This implies that there may be a dose‐dependent relationship between AAS use and myonuclei density, but the effect of cycling on and off AAS on such irreversible biomarkers remains unclear with these findings [18]. Overall, AAS, testosterone, and other similar androgen receptor activating substances will efficiently increase muscle hypertrophy in a supraphysiological manner, leaving additional myonuclei, SCs, and increased androgen receptor density as potentially the only architectural biomarkers of prohibited substance use following cessation. It is still unclear what other classes of drugs have this potential on muscle tissue and if their lasting impact translate to performance enhancement.

## 7 Epigenetic Changes

In addition to the myonuclear permanence that serves as a physiological imprint on muscle fibers, researchers have explored genetic and epigenetic modifications at a cytoarchitectural level. This is demonstrated by alterations in gene expression as a result of nongenetic structural changes to DNA or histones (referred to as methylation) resulting in altered access to transcriptional equipment primarily by increasing or decreasing RNA polymerase activity [40, 41]. DNA/histone methylation involves the chemical bond between a covalent methyl (CH_3_) group and the 5th position of the pyrimidine ring of a cytosine (C) nucleotide, resulting in 5‐methylcytosine (5mC) [40, 41]. Typically, this occurs on the cytosine nucleotide where it is followed by a guanine nucleotide (G) and separated by a phosphate (“p”) group within the same strand of DNA, referred to as a CpG site [40]. The methylation process leads to changes in gene expression when CpG methylation recruits CpG methyl binding proteins that inhibit gene transcription [40]. Therefore, increased CpG methylation leads to reduced gene expression and decreased CpG methylation leads to increased gene expression [40]. Resistance exercise has been predominately confirmed as a hypomethylating stimulus, explaining its ability to increase MPS and overall protein turnover [40, 41].

Seaborne et al., in 2018, first investigated the theory that DNA hypomethylation occurs during a period of resistance training, remains after a subsequent period of detraining, and persists into a period of retraining [40, 42]. The researchers found that DNA retained some but not all of its epigenetic modifications [40, 42]. Among the genes that retained epigenetic modifications, some showed a larger magnitude of change in DNA hypomethylation when exposed to retraining, suggesting that the original training period may have acted as a “primer” for an enhanced response to the reintroduction of training [40, 43]. Interestingly, these changes were associated with increased transcriptional expression of the same genes correlated with the increase in lean muscle mass seen upon retraining (namely, UBR5, RPL35a, HEG1, PLA2G16, and SETD3) [40, 43]. These findings have been repeated in rodent models and human populations [28, 41]. While the impact of AAS on both anabolic and catabolic epigenetic markers has been explored largely in rodent models, ongoing research aims to confirm similar markers and potentially identify new ones in previous human AAS users. A systematic review by Pelton et al. (2023) concluded that various AAS were most often linked to the upregulation of the anabolism related genes: MYOG, MyoD, and IGF—responsible for skeletal muscle cell differentiation and proliferation processes) significantly greater than controls [44]. In contrast, these substances commonly saw the upregulation of the catabolism related gene: MSTN—responsible for suppressing myogenesis and SC activity. While these findings may not aid in the understanding of signaling pathways modified by AAS, they may indicate other potential biomarkers that could be investigated for past AAS use. Other genetic and epigenetic markers extending outside of those related with skeletal muscle include markers of oxidative stress, and endocrine/immune function have also been suggested [45, 46].

## 8 Fiber Type Differences

When referring to hypertrophy‐stimulating or resistance exercise, the focus is often on glycolytic, type II (fast‐twitch) muscle fibers, dominant in power‐driven exercises, and intense weight training. It has also been shown that type II fibers generally have higher level of androgen receptor expression, making them a more susceptible target of AAS binding [47]. However, it is important to consider that while many athletes using AAS come from strength and power disciplines, they may also be abused in aerobically dominant disciplines such as cycling, swimming, and track and field. The lasting effect of AAS on muscle tissue under these circumstances is still unclear. Endurance exercise is generally measured through metabolic adaptations such as fatigue resistance rather than through cytoarchitectural changes. Consequently, accretion of myonuclei or hypomethylation may have no lasting performance benefit in these settings.

It is well‐known that there is greater metabolic activity and muscle protein turnover rates in type I (oxidative) compared to type II muscle fibers [29, 48]. Oxidative muscle fibers experience preferential SC accumulation and myonuclear accretion compared with glycolytic fibers during hypertrophy induced by bicycle ergometer training in humans, indicating potential fiber‐type differences in SC requirements [39, 48]. Bruusgaard et al. (2003) confirmed this when they found the number of nuclei in fibers of the same size is higher in oxidative than glycolytic fibers, attributed to the higher turnover demands in oxidative muscle [39, 49]. For endurance athletes, they will likely display a greater number of oxidative rather than glycolytic fibers accompanied by a greater number of SCs and myonuclei compared to their glycolytic counterparts. However, as the myonuclear domain theory has been explained thus far, it is unlikely that myonuclear permanence would apply to oxidative muscle in the same way it does glycolytic muscle. Conditioning oxidative muscle typically results in increased oxidative metabolism (mitochondrial density) rather than hypertrophy [49]. It remains unclear whether SC and myonuclei accumulation would benefit and endurance performance or aid in returning to endurance activity after a period of detraining. This is an important consideration for endurance athletes, especially in scenarios where the rapid return to a specific pace in a race is crucial, as SCs and myonuclei are associated with the synthesis of proteins essential for muscle building.

## 9 Sex Differences

Predictably, there has been a lack of research on the differences in muscle memory between sexes. While we can infer the impact of endogenous and exogenous hormones on muscle mass accumulation and note the natural differences in fiber type dominance between males and females, there has been no direct comparison on these parameters. Drawing from existing data, such as the fact that males generally have around 30% more muscle mass and strength than females, and considering that females exhibit a higher rate of MPS at rest, we can make potential deductions [50, 51, 52]. Though it was previously shown that females carry predominately Type I muscle fibers and males carry predominately Type II—we now see the two sexes to be more similar in fiber type distribution than different. However, it stands that MPS rates are known to be higher in Type I fibers due to the turnover demands of oxidative tissue [23]. Taking these factors into account, it is conceivable that males, with their higher muscle mass and strength, may have a larger surface area for myonuclei, potentially allowing for faster production of new myonuclei compared to females [52]. On the other hand, highly trained females, with their higher relative myonuclei density and SC numbers, may benefit from the turnover demands of oxidative tissue [48, 51]. When examining the effects of AAS on muscle profiles, both males and females may undergo muscle hypertrophy and enhanced athletic performance (though the available studies involving females were performed with animal subjects) [32, 33]. However, the effectiveness of AAS is influenced by the availability of androgen receptor sites. Typically, males have a greater number of androgen receptors, leading to more pronounced anabolic effects due to higher baseline testosterone levels and androgen receptor density [51]. Relating this back to muscle memory and the theory of myonuclear density, it is plausible that males, using AAS under similar conditions, may recruit a greater absolute number of SC and new myonuclei than females, though further confirmation is needed.

## 10 Putting it all Together

In conclusion, the phenomenon of muscle memory, marked by myonuclear permanence and potential genetic and epigenetic modifications, presents intriguing aspects in the context of exercise, training, and AAS use. The myonuclear domain theory suggests that myonuclei acquired during hypertrophy may remain stable or even permanent, contributing to enhanced MPS and potential benefits in muscle memory. Continuous training without a period of detraining is an area that warrants further investigation, especially in antidoping efforts, as most athletes subjected to a doping sanction would not be expected to detrain. Moreover, the impact of performance enhancing drugs (other than AAS) such as growth hormone secretagogues, beta‐2 agonists, IGF‐1 peptides, or hormone and metabolic modulators on muscle tissue plasticity are seldom researched and still raise questions about potential long‐term effects, even after cessation. The resilience of myonuclei through various muscle wasting conditions implies that while skeletal muscle fiber size may decrease post‐PED use, SC activity, myonuclear density, and neurological connectivity may persist. The influence of sustained training, protein intake, and resistance exercise on muscle memory, particularly in elite athletes, requires careful consideration. The fiber‐type differences in oxidative and glycolytic muscle fibers, along with potential variations in SC and myonuclei response in these two muscle fiber types, pose challenges in understanding the applicability of muscle memory to endurance performance and recovery.

Additionally, the exploration of genetic and epigenetic modifications, particularly DNA methylation, provides insights into the potential role of these factors in muscle memory. Hypomethylation induced by resistance exercise may contribute to increased MPS and could play a role in the enhanced response to retraining, as observed in various studies. In the realm of antidoping efforts, understanding the complexities of muscle memory becomes crucial. Continuous training coupled with AAS/PED use and/or cessation, and the interplay of genetic and epigenetic factors offer avenues for further research to better leverage muscle memory in maintaining performance gains and addressing potential challenges in the fight against doping in sport.

## Author Contributions

C.T. conducted the literature review motivating the content in this manuscript and wrote the manuscript.

## Disclosure

C.T. was employed by the World Anti‐Doping Agency (WADA) at the time this manuscript was drafted, submitted and revised.

## Conflicts of Interest

C.T. was employed by the World Anti‐Doping Agency (WADA) at the time this manuscript was drafted, submitted, and revised. This is being declared as a potential conflict of interest. The draft manuscript was sent to current employees of WADA for review. C.T. was employed by WADA at the time the of manuscript's submission.
